# Altered functional connectivity of the default mode and frontal control networks in patients with insomnia

**DOI:** 10.1111/cns.14183

**Published:** 2023-03-21

**Authors:** Hui Zheng, Qin Zhou, Junjie Yang, Qian Lu, Huaide Qiu, Chuan He, Hailang Yan

**Affiliations:** ^1^ Department of Rehabilitation Medicine The Affiliated Jiangsu Shengze Hospital of Nanjing Medical University Suzhou Jiangsu China; ^2^ Shanghai Key Laboratory of Psychotic Disorders, Shanghai Mental Health Center Shanghai Jiao Tong University School of Medicine Shanghai China; ^3^ Rehabilitation Medicine Center The First Affiliated Hospital of Nanjing Medical University Nanjing Jiangsu China; ^4^ Department of Radiology The Affiliated Jiangsu Shengze Hospital of Nanjing Medical University Suzhou Jiangsu China

**Keywords:** fMRI, functional connectivity, insomnia, repetitive transcranial magnetic stimulation

## Abstract

**Aims:**

The purpose of this study was to investigate the association between spontaneous regional activity and brain functional connectivity, which maybe can distinguish insomnia while being responsive to repetitive transcranial magnetic stimulation (rTMS) treatment effects in insomnia patients.

**Methods:**

Using resting‐state functional magnetic resonance imaging data from 38 chronic insomnia patients and 36 healthy volunteers, we compared the amplitude of low‐frequency fluctuations (ALFF) between the two groups. Of all the patients with insomnia, 20 received rTMS for 4 weeks, while 18 patients received a 4‐week pseudo‐stimulation intervention. Seed‐based resting‐state functional connectivity (RSFC) analysis was conducted from regions with significantly different ALFF values, and the association between RSFC value and Pittsburgh Sleep Quality Index score was determined.

**Results:**

Our results revealed that insomnia patients presented a significantly higher ALFF value in the posterior cingulate cortex (PCC), whereas a significantly lower ALFF value was observed in the superior parietal lobule (SPL). Moreover, significantly reduced RSFC was detected from both PCC to prefrontal cortex connections, as well as from left SPL to frontal pole connections. In addition, RSFC from frontal pole to left SPL negatively predicted sleep quality (PSQI) and treatment response in patients' group.

**Conclusion:**

Our findings suggest that disrupted frontoparietal network connectivity may be a biomarker for insomnia in middle‐aged adults, reinforcing the potential of rTMS targeting the frontal lobes. Monitoring pretreatment RSFC could offer greater insight into how rTMS treatments are responded to by insomniacs.

## Graphical abstract

The goal of this study was to use rs‐fMRI to investigate the brain underpinnings of insomnia. We discovered that the brain regions with significantly lower spontaneous activity (fALFF) were located in the superior parietal lobule, that the functional connectivity with the frontal pole was significantly reduced, and that it was especially related to sleep problems.

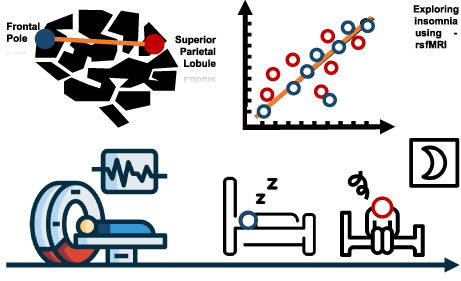

## 1 INTRODUCTION

Sleep issues, like insomnia, are often found as comorbidities alongside other conditions such as Parkinson's disease, chronic pain, anxiety disorders, depressive disorders, and substance abuse disorders.^1^ The identification of factors which can predict treatment response can be beneficial in the designing of new treatment strategies, in addition to helping to advance personalized medicine within psychiatry. Psychiatric symptoms are caused by dysregulated dynamic cross‐network interactions between the salience (SN), frontoparietal network (FPN, also known as central executive network), and default mode networks (DMNs).^2^ Alterations in resting‐state brain activity are not only considered to be a consequence of insomnia but also changes in brain networks that maintain insomnia.^3^ Primary insomnia is the most typical sleep disorder, which is associated with substantial impairment in quality of life,^4^ and the global prevalence of insomnia is between 10% and 15%.^5^ During COVID‐19, the problem has become even worse among older people, with 24.4%–26.8% of Chinese adults aged ≥60 years experiencing insomnia in the last month.^6^ With the limitations of both pharmacological^7^ and cognitive‐behavioral therapy,^8^ there is a crucial need for the development of effective, safe, and accessible insomnia treatment options. To optimize treatment outcomes, a potential strategy could be to identify pretreatment neural predictors of treatment response, so as to establish which patients are likely to respond to a given treatment. However, one potentially effective way of searching for biomarkers of treatment outcomes may be to first explore intermediate phenotypes via diagnostic neuroimaging.^9^

Noninvasive brain stimulation, such as repetitive transcranial magnetic stimulation (rTMS), has been shown to be safe and has the potential to improve insomnia in different types of neurological and neuropsychiatric disorders.^10^ Initially, the first study has reported that rTMS can improve subjective sleep quality in depression patients.^11^ Moreover, several studies have reported that rTMS can modulate arousal,^12^ sleep quality,^13^ and sleep‐related plasticity.^14^ Patients with chronic insomnia show abnormal low‐frequency fluctuations (ALFF) in several subregions of the DMN and dorsal attention network (DAN), and ALFF values were positively correlated with the severity of insomnia.^15^ The potential mechanism by which rTMS improves sleep in patients with insomnia might involve a hyperarousal model in the cerebral cortex^16^ through anatomical and functional connectivity, affecting metabolic activity^17^ and hormones^18^ associated with sleep. Additionally, noninvasive techniques of neurostimulation may be an effective way to reduce cognitive decline associated with aging and neurodegeneration.^19^

However, there is a lack of biomarkers to predict the effectiveness of rTMS treatments in insomnia. Could resting‐state spontaneous brain activity as a consequence of insomnia and as a potential maintenance mechanism predict the brain response to rTMS intervention? Resting‐state functional magnetic resonance imaging (fMRI) not only provides neural processing information that may serve as a potential target but is also easy to operate in a clinical setting, such as with the application of resting‐state functional connectivity (RSFC).^20^ The first step in RSFC analysis is to select a region of interest (ROI) for seed‐based analysis based on prior assumptions. Low‐frequency (usually 0.01–0.08 Hz) fluctuations are a steady index for spontaneous activity^21^ and can help in the identification of suitable ROI. Subsequently, the ALFF has been introduced to detect altered brain states in various diseases, including Alzheimer's disease,^22^ schizophrenia,^23^ and insomnia^15^ Specifically, ALFF altered associated with major psychiatry disease is widely distributed in several DMN subregions.^15^ However, as these results point to the DMN, is it the local activity of the DMN or the RSFC from the DMN that predicts treatment outcome?

This study aimed to identify a resting‐state fMRI biomarker for insomnia. Benzodiazepines and other sedative‐hypnotic drugs are prescribed to many older people despite a nearly fivefold increase in the risk of adverse cognitive events associated with them.^24^ Furthermore, the accumulation of side effects from long‐term medication use is an important issue in the management of older people with insomnia,^25^ and rTMS might be used as a potential tool to address this issue.^26^ Previous studies have demonstrated that insomnia patients often also have depression and anxiety, and frontoparietal reticular dysfunction is associated with disease duration and anxiety.^27^ We hypothesized that (1) insomnia patients on medications would show more alert ALFF in the DMN and FPN than healthy participants, and (2) the RSFC of the alerted brain region would predict insomnia severity. By calculating ALFF and RSFC across various brain regions using correlation analysis, we tried to test these two hypotheses. Finally, because treatment adherence is a known predictor of rTMS response,^28^ we examined whether spontaneous brain activity in those who discontinued treatment differed from spontaneous brain activity in those who continued treatment to control for treatment effects.

## 2 METHODS

### 2.1 Participants

Forty‐five right‐handed older adult patients with chronic insomnia and 41 age‐ and education‐matched healthy older adult volunteers without insomnia were included in this study from July 2020 to July 2021. Patients were recruited from the Affiliated Jiangsu Shengze Hospital of Nanjing Medical University, Outpatient Department of Rehabilitation, and healthy volunteers were recruited from the Wujiang Shengze Zhen Old‐age University. Inclusion criteria for the patients were (1) patients who met the diagnostic criteria of primary insomnia of the Diagnostic and Statistical Manual of Mental Disorders, Fourth Edition^29^; (2) patients aged 45–75 years; (3) patients with the educational level of junior high school or above; (4) patients who agreed to participate voluntarily in the experiment. Exclusion criteria for both groups were current neurological or other psychiatric diseases. Participants did not report a history of head injury, neurodevelopmental disorders (intellectual disability), neurocognitive diseases (dementia), or other sleep disorders (obstructive sleep apnea [OSA], restless legs syndrome [RLS], periodic leg movements [PLM], REM sleep behavior disorder [RBD]). The above sleep‐related disorders are mainly diagnosed using the ICD‐10 China version for exclusion and structural MRI. Participants with substance use or other addictive disorders were also excluded from the study. The demographics and clinical characteristics of all subjects are shown in Table 1. Patients had a history of chronic, heavy, and repeated use of sleep aids, alprazolam, eszopiclone tablets, etc., and a high rate of insomnia relapse after the withdrawal of medication. Jiangsu Shengze Hospital of Nanjing Medical University Affiliated Ethics Committee approved the study (JSSZYY‐LLSC‐202018). All participants underwent MRI after they provided written informed consent. Data from 38 patients and 36 healthy volunteers met our quality control requirements after preprocessing (the details are shown in Section 2.4) and were included in subsequent analyses. A total of 20 patients completed the treatment. The registration number is ChiCTR2100049455 (Chinese Clinical Trial Registry), and the registry name is “Application of neurodegenerative techniques for insomnia and cognitive impairment in the elderly.”

**TABLE 1:** Demographics of all participants in this study.

	Insomnia *n* = 45	HV *n* = 41	*t*/*χ* ^2^	*p*	Cohen's *d*
Gender (male)	14	10	0.49	0.481	/
Age (years)	56.00 ± 10.50	58.90 ± 8.40	1.482^a^	0.142	0.310
Education year	8.85 ± 4.40	9.88 ± 8.85	−1.297	0.198	0.274
FTND score	0.19 ± 0.85	00.05 ± 0.21	1.130^a^	0.263	−0.229
AUDIT score	0.75 ± 2.16	00.19 ± 0.79	1.654^a^	0.103	−0.337
BMI	22.24 ± 2.64	23.10 ± 2.33	1.500	0.138	0.349
Insomnia year	3.60 ± 4.10	/	/	/	/

Abbreviation: HV, healthy volunteer.

^a^ Levene's test is significant (*p* < 0.05), and Welch's *t* test was used.

### 2.2 Measurement of sleep quality

Sleep quality was assessed using the Pittsburgh Sleep Quality Index (PSQI), which is a self‐report survey that comprises 19 items across seven components to generate a global score and can be completed in 5–10 min.^30^ As a standardized sleep questionnaire, the PSQI was designed to be used by clinicians and researchers with ease and has been used in various populations. The 19 items measure various aspects of sleep to provide seven separate component scores, as well as a composite score. The components are subjective sleep quality, sleep latency (the amount of time it takes to fall asleep after the lights have been turned off), sleep duration, habitual sleep efficiency (the proportion of sleep time spent awake), sleep disturbances, use of sleeping medications, and daytime dysfunction. Each item is rated from 0 to 3. The total of the seven component scores is the global PSQI score, ranging from 0 to 21, where lower scores indicate better sleep quality. The PSQI was administered before (pre), after (post) the treatment, and at follow‐up (1 month later).

### 2.3 Repetitive transcranial magnetic stimulation treatment

**FIGURE 1:**
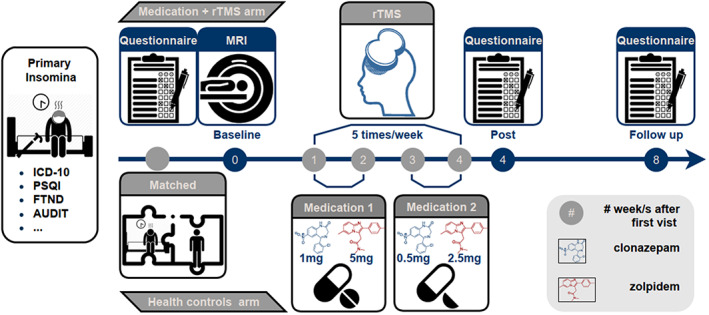
Experimental flow chart. Patients with primary insomnia were recruited through outpatient clinics, excluding those with other psychiatric disorders. Twenty of these patients completed pharmacological treatment combined with a transcranial magnetic stimulation intervention five times a week. In addition to self‐reports of sleep quality and resting‐state MRI measurements, a cohort of healthy subjects was recruited prior to the intervention. After the intervention and 8 weeks later, self‐reports of sleep quality were completed.

RTMS was performed using a MagPro device (MagPro X100, Tonica Elektronik A/S) connected to a figure‐8 coil. The coil was oriented over the right dorsolateral prefrontal cortex (dlPFC), with a horizontal angle of 45° relative to the nasion‐inion midline. Magnetic pulses were delivered at a frequency of 1 Hz at an intensity of 90% of the motor threshold, with 40 trains of 30 s on and 8 s off. Sessions were conducted five times per week for 4 weeks at 1200 pulses per session. During the rTMS treatment period, 20 patients' medications followed two phases: the first phase (weeks 1–2) consisting of clonazepam 1 mg and zolpidem 5 mg (2.5 mg daily at bedtime [qhs]) and the second phase (weeks 3–4) consisting of clonazepam 0.5 mg and zolpidem 2.5 mg qhs. These two drugs are commonly used to treat primary insomnia. The combination of these two drugs may increase the risk of falls in older people, but they can also be used in controlled doses to maintain sleep quality. Zolpidem is a non‐benzodiazepine sedative and is one of the first‐line drugs recommended by current guidelines for inducing sleep latency. However, it has a very short half‐life and may cause early awakening. In contrast, clonazepam is a benzodiazepine with a long half‐life of over 10 h and a long duration of action, but it has a slow onset of action of 1–2 h. The use of clonazepam together with zolpidem may increase related side effects, such as dizziness, drowsiness, confusion, and poor concentration. No patient in this study reported more than one significant side effect (due to either medication or rTMS) through retrospective verbal questioning at the revisit (4‐ or 8‐week follow‐ups). For the sham group, only medication was used and no rTMS intervention was performed (Figure 1).

### 2.4 MRI acquisition and preprocessing

All subjects underwent an MRI scan on a 3.0T GE Discovery MR750w scanner while in a head‐first supine position for 10 min for the resting‐state scan and 5 min for the structural scan before starting the treatment. A gradient‐echo echo‐planar imaging T2* sensitive pulse sequence was used to acquire resting‐state fMRI data (interleaved sequence, 41 slices, 3.5‐mm thickness, 3 × 3‐mm pixel spacing, 2500‐ms repetition time [TR], 30‐ms echo time [TE], 192 × 192‐mm field of view [FOV], 90° flip angle, and 64 × 64 acquisition matrix). A three‐dimensional, spoiled‐gradient recalled T1‐weighted sequence (axial T1 BRAVO) was used to acquire whole‐brain structural data with an acquisition time of 301 s (188 slices, 1‐mm thickness, TR = 8692 ms, TE = 3.2 ms, skip = 0 mm, 12° flip angle, inversion time = 450 ms, FOV = 256 × 256 mm, and 256 × 256 acquisition matrix).

Data Processing Assistant for Resting‐State fMRI (DPARSF) version 5.2 (http://rfmri.org/dparsf) was used for the preprocessing of the fMRI data. It was based on the SPM software package version 12 (http://www.fifil.ion.ucl.ac.uk/spm). The first 10 volumes were discarded to allow the magnetization to reach a dynamic equilibrium and allow participants to get used to the scanning noise. Slice‐timing, reorientation, and realignment to the first volume were performed, followed by T1 co‐registration. DARTEL was used to segment the skull‐stripped T1 images. Then, nuisance covariate regression was performed. A 24‐parameter Friston model was used to correct for motion.^31^ Subjects were excluded if translations or rotations of the head exceeded 2.0 mm, which resulted in the exclusion of seven patients (7/45) and five healthy volunteers (5/41). Physiological artifacts were reduced by combining cerebrospinal fluid, white matter, and global signals. The DARTEL tool was used to compute transformations from individual native space to Montreal Neurological Institute space of 3 × 3 × 3 mm^3^.^32^

### 2.5 ALFF and RSFC calculations

DPARSF was used to calculate ALFF. The filtered time series of each voxel was transformed into a frequency domain and subsequently into a power spectrum using fast Fourier transform. The square root of the signal over 0.01–0.08 Hz was measured in each voxel. For spatial smoothing, a 4‐mm full‐width half‐maximum Gaussian kernel was used. Based on the results of the ALFF analysis, we chose the bilateral posterior cingulate cortex (PCC) and superior parietal lobule (SPL) from the AAL (90 cortex regions) template as seeds. Seed‐based RSFC was calculated using DPARSF, and the signal value in the PCC and SPL was exact. The confounding signals related to white matter and cerebrospinal fluid were removed using linear regression. Fisher's z‐transform was used to convert correlation coefficients to *z*‐values to improve the normality of the distribution.

### 2.6 Statistical analysis

Continuous variables with normal distributions were presented as means and standard deviations, with logarithmically converted model parameters. For continuous variables, we also performed tests of normality. Group comparisons were conducted using independent‐sample *t*‐tests (normal distribution) or Mann–Whitney *U*‐test (non‐normal distribution) for continuous variables and chi‐squared tests for categorical variables. The fMRI results were corrected for multiple comparisons using the Gaussian random field, with voxel and cluster significance thresholds set to *p* < 0.001 and *p* < 0.05, respectively (*t* > 3.20). We analyzed and visualized the *z*‐scores of RSFC in the significant regions (i.e., the dlPFC, inferior frontal gyrus [IFG], and frontal pole [FP]) with the dabest package (version 0.26).^33^ A cluster was created from the remaining surviving voxels that were correlated with the dlPFC. The clusters were then extracted and imported into the R‐based statistical software jamovi, version 1.8.4 (www.jamovi.org). We employed the Pearson correlations between baseline sleep quality (PSQI score), treatment effect (pre‐PSQI score − post‐PSQI score), ALFF value, and RSFC value. We also use the RSFC as predict variable, the treatment effect as predicted variable in a linear regression model to investigate can the RSFC distinguish insomnia while being responsive to rTMS treatment effects. Significant results were defined by *α* < 0.05. Bonferroni correction (*α*/*m*, where *m* = the number of comparisons) was applied to correct for multiple comparisons between neuroimaging and questionnaire data.

## 3 RESULTS

### 3.1 Difference in ALFF between insomnia patients and healthy volunteers

**FIGURE 2:**
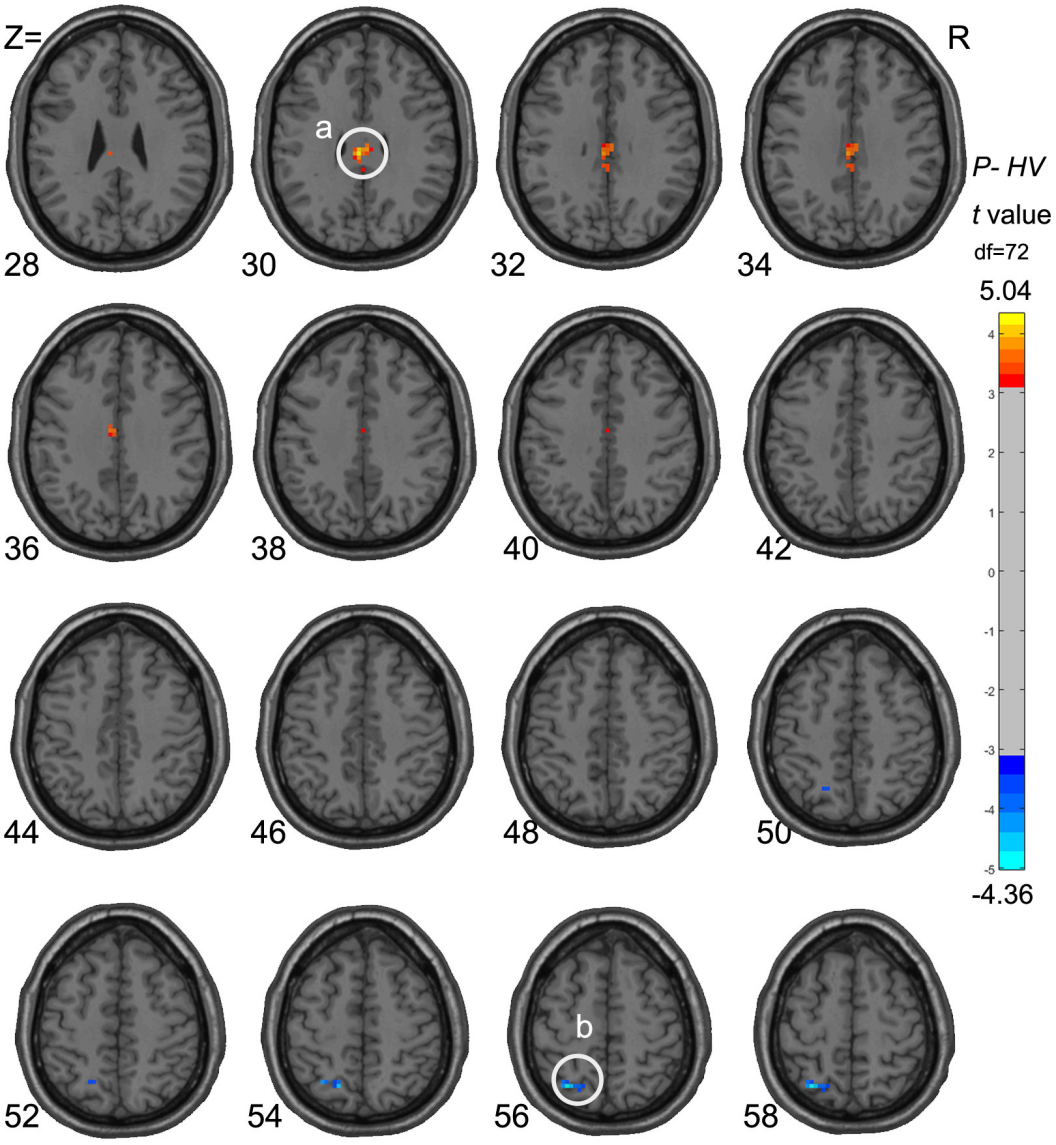
Difference in ALFF value between insomnia patients and healthy volunteers. The color bar represents *t*‐values (healthy volunteers–insomnia patients). Those with chronic insomnia had higher ALFF values in the PCC (A) and lower ALFF values in the left SPL (B).

The significant main effect of the groups showed that patients with primary insomnia had higher ALFF values in the PCC (*x* = −3, *y* = −24, *z* = 30, *k* = 33, max *t* = 5.04) and lower ALFF values in the SPL (*x* = −30, *y* = −63, *z* = 57, *k* = 18, max *t* = −4.36) than healthy volunteers. Detailed results are provided in Figure 2.

### 3.2 Difference in RSFC between insomnia patients and healthy volunteers

**FIGURE 3:**
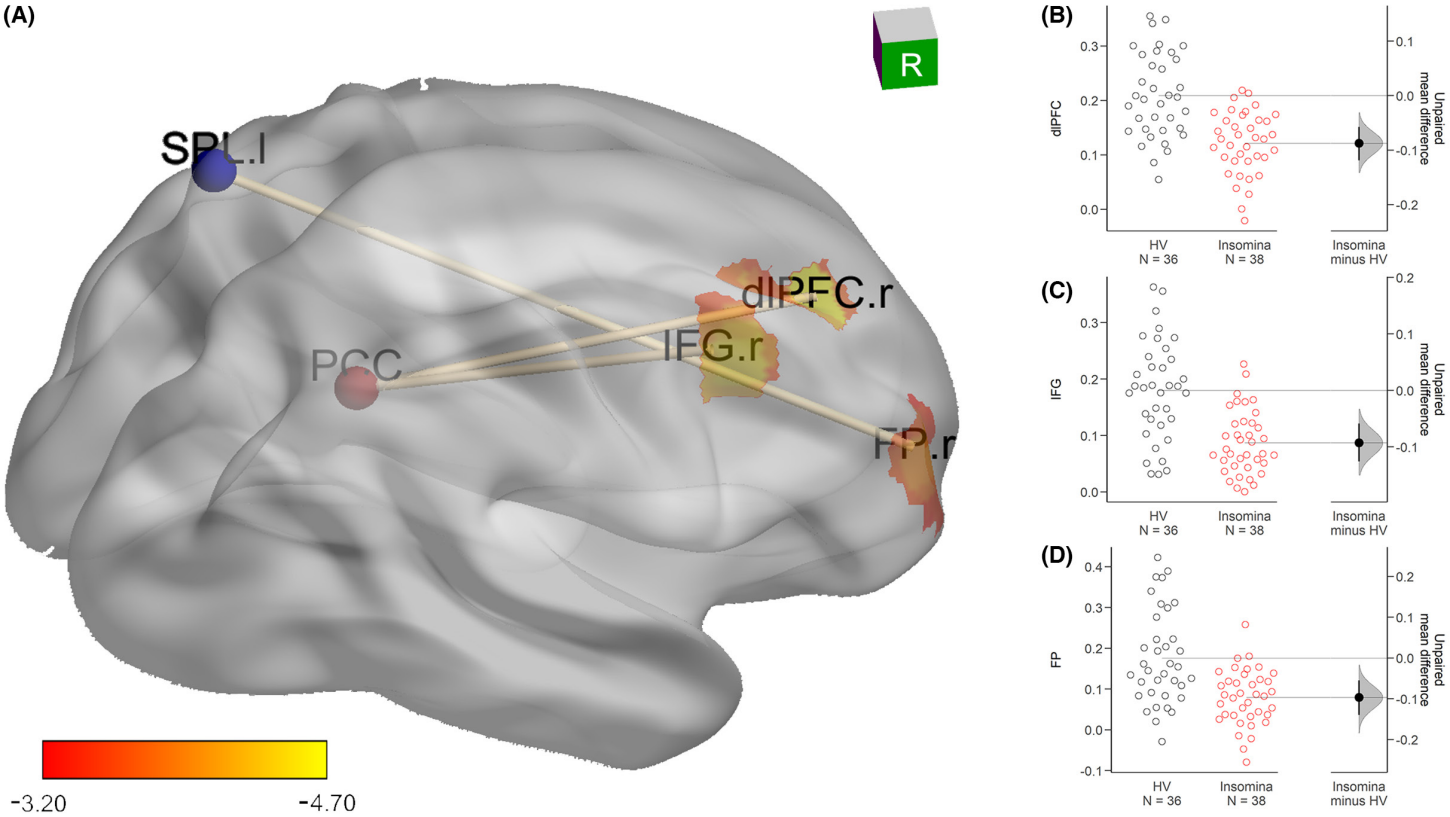
RSFC difference between insomnia patients and healthy volunteers. The brightness of the color represents *t*‐values (healthy volunteers–insomnia patients): brighter colors indicate higher absolute values. In patients with chronic insomnia, RSFC from the PCC to the right IFG and right dlPFC was significantly lower than that of healthy volunteers. The RSFC value from the SPL to the FP was also significantly lower than that of healthy volunteers. Extracted *z*‐values of the corresponding brain regions were compared by unpaired *t*‐tests, which are visualized as a normal distribution scatterplot on the right. The black solid point is the mean difference between the two groups, the black line is the 95% confidence interval of the mean, and the shaded regions are the probability of the distribution.

For the PCC seed, compared with healthy volunteers, patients with primary insomnia had lower RSFC with the frontal regions, which included the dlPFC (*x* = 24, *y* = 36, *z* = 39, *k* = 31, max *t* = −4.70) and IFG (*x* = 36, *y* = 12, *z* = 39, *k* = 34, max *t* = −4.70). For the SPL seed, compared with healthy volunteers, patients with chronic insomnia had lower functional connectivity with the FP (*x* = 33, *y* = 51, *z* = 9, *k* = 43, max *t* = −4.00). Further details are provided in Figure 3.

### 3.3 Correlation between RSFC and sleep quality

**FIGURE 4:**
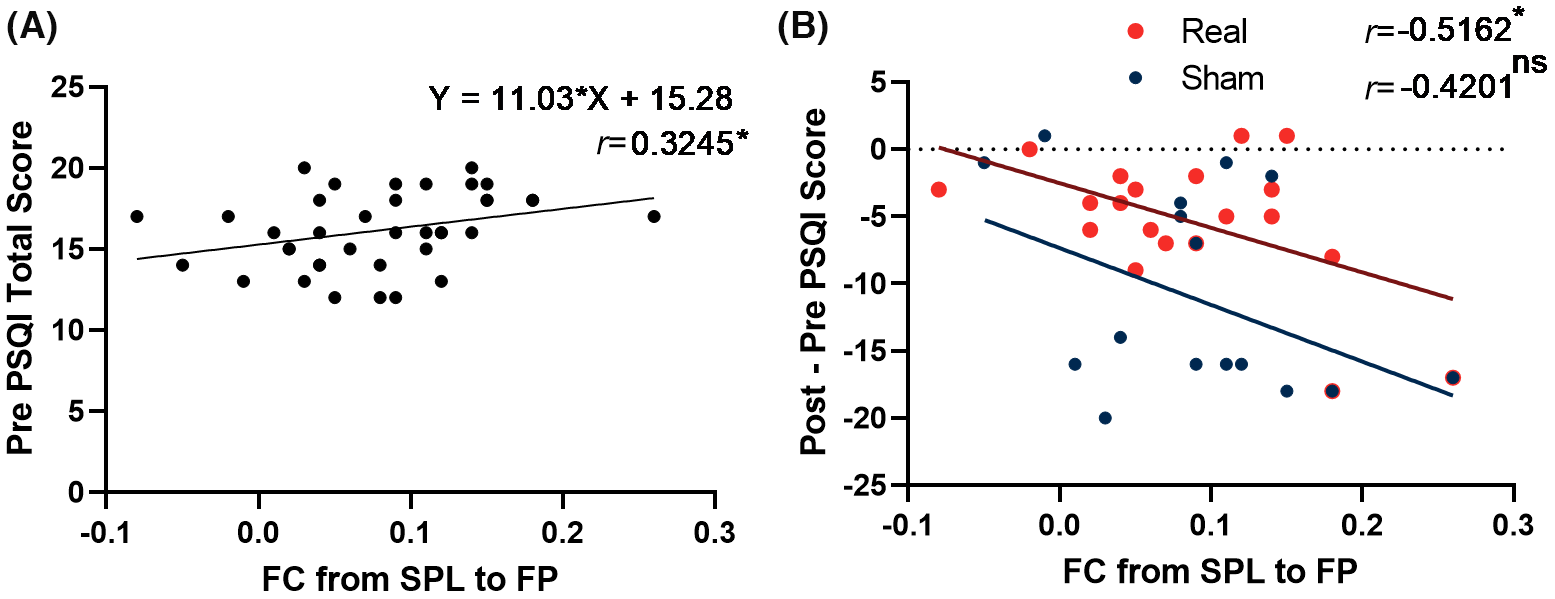
Insomnia symptoms were correlated with RSFC values of the SPL to FP. (A) RSFC value at baseline was significantly correlated with baseline PSQI. (B) The treatment effect (Post–Pre total score) was significantly positively correlated with the baseline RSFC value in real stimulate condition rather than the sham condition. The rTMS treatment effect was measured by subtracting the pretreatment PSQI from the post‐treatment PSQI.

Pearson correlation analysis and linear regression showed that the RSFC value between the SPL and FP was positively correlated with the baseline PSQI score (*N* = 38, *r* = 0.32, *p* = 0.047, with *Y* = 11.03 * *X* + 15.28, Figure 4A) and change in PSQI score (for real group post‐pre: *N* = 20, *r* = 0.52, *p* = 0.02, with *Y* = −33.17 * *X* − 2.514; for sham group: *N* = 18, *r* = 0.42, *p* = 0.081, with *Y* = −42.23 * *X* − 7.345. Figure 4B). The following analysis between the group of withdrawal and compliance did not show any significant differences in each index. This might mean that compliance did not play a role in this study. No correlations were found between the PSQI score and ALFF value or RSFC seeded from the PCC.

## 4 DISCUSSION

The purpose of this study was to investigate fMRI biomarkers that could distinguish insomnia and be responsive to rTMS treatment effects. Three significant findings were identified when comparing chronic insomnia patients to healthy volunteers. Firstly, ALFF values were found to be significantly higher in the PCC and lower in the SPL in insomnia patients as compared to healthy volunteers. Secondly, patients exhibited significantly lower RSFC between PCC/SPL and the prefrontal cortex. Finally, the RSFC between the SPL and FP predicted the treatment effect of rTMS. These results lend support to our first hypothesis that abnormal spontaneous brain activity exists in DMN and FPN regions of insomnia patients. While the second hypothesis was only partially supported, our findings suggest that sleep problems can be primarily predicted through functional connectivity within the FPN. These findings contribute to the identification of a potential biomarker for primary insomnia and highlight the potential of rTMS in targeting the frontal lobes in middle‐aged and older adults.

The ALFF within the DMN and FPN may allow for distinguishing insomnia patients from healthy volunteers. We identified the lower ALFF in the left SPL in insomnia patients, which might be related to the impairment associated with insomnia. The SPL is included in the FPN and DAN and is involved in the spatial orientation function, which enables individuals to remember the location of objects in space and their visual and tactile characteristics.^34^ Moreover, it plays an important role in the manipulation of information and resetting of working memory.^35^ One electroencephalography study has shown that women with lower vigilance had higher activity in the right SPL.^36^ Morphological studies have also reported that primary insomnia patients showed cortical thickening in the left SPL, bilateral insula, and left middle cingulate cortex.^37^ The PCC is a central node in the DMN, simultaneously communicates with various brain networks (e.g., FPN and DAN), and participates in numerous brain functions (e.g., autobiographical memory retrieval, self‐referential processing, interoception, or imagining the future).^38^ We found that patients with primary insomnia had significantly higher ALFF in PCC than healthy volunteers. One study has shown that, compared with healthy controls, core DMN regions in insomnia patients showed greater activation in self‐reference‐related tasks.^39^ In another study, insomnia patients had higher dynamic ALFF in the bilateral hippocampus (including the right insula and putamen), and that correlation was associated with self‐reported anxiety.^40^ The results of this study demonstrated the potential of ALFF as a diagnostic biomarker by detecting changes in status more accurately.

The RSFC between the DMN and FPN may help differentiate between patients with insomnia and healthy volunteers. Several studies have shown that long‐term chronic insomnia can cause damage to brain networks,^41^ with DMN being the most commonly reported.^15^ Previous studies have reported that compared with healthy controls, patients with primary insomnia show higher RSFC between the left insula and the right anterior cingulate cortex^42^; higher RSFC between the premotor cortex and sensorimotor cortex and lower RSFC between the amygdala, insula, striatum, and thalamus^43^; higher RSFC within the limbic network but lower RSFC within the DMN^44^; and higher RSFC between the bilateral middle and left middle frontal gyrus.^45^ Our results found that this alteration was not highly correlated with insomnia, which is similar to previous studies. One study has shown that damage to RSFC between the DMN and supplementary motor area was correlated with earlier onset of insomnia.^46^ The seed‐based region‐to‐region RSFC method has demonstrated that insomnia patients showed significantly decreased functional connectivity between the medial prefrontal cortex and right medial temporal lobe and between the left medial temporal lobe and left inferior parietal cortex, which are included in the DMN.^47^ Another network analysis has also revealed that insomnia patients have decreased RSFC between the anterior and posterior DMN and increased RSFC between the FPN and DAN.^39^

In insomnia patients, impairment in the FPN may serve as a potential predictor of treatment response to rTMS. Specifically, we discovered a positive association between the severity of insomnia and RSFC between the FP and SPL. Notably, the FPN has been widely implicated in resting‐state brain network abnormalities in insomnia. A meta‐analysis has shown that executive control impairment in insomnia patients is mild to moderate.^48^ One study using the independent component analysis has found that the RSFC of the right FPN in patients with primary insomnia was decreased.^27^ Another study has demonstrated less functional connectivity variability between the anterior SN and left FPN.^49^ Furthermore, the effect of drugs combined with TMS treatment was positively correlated with the connectivity strength of the FPN, indicating that rTMS may be primarily influenced by FPN plasticity. First, our stimulation targets were located in the prefrontal lobe. Second, stimulation of the prefrontal lobe affects a wide range of parietal and subcortical areas.^50^ The central nervous system regulates sleep homeostasis through cytokine responses that link sleep to the immune system.^51^ A potential direction of interpretation is that rTMS might enhance sleep quality in insomnia patients through neuroendocrine and autonomic pathways. However, this hypothesis needs to be explored more.

The study had several limitations that need to be considered. First, our patients were recruited from a population that mainly comprised individuals who were in good health and had high socioeconomic status. This might have introduced bias in our study because this sample likely paid more attention to the quality of life and had a high level of social support. Thus, prospective large samples and differentiation of sleep phenotypes and subtypes are required to add information on related genetics. Second, the small sample size of the treatment group did not allow for robust results to predict the therapeutic effect of rTMS. It is true that our results were not focused on the prediction of response to treatment; hence, the description of treatment was not detailed but provided an exploratory map in the results section (Figure 4B). However, the RSFC has the potential to be a predictor of treatment effect. Thus, future exploratory research will require larger samples. Third, the lack of a sham group for baseline comparisons limited our explanation of the therapeutic effect of rTMS for insomnia. Fourth, the PSQI questionnaire, although it is an effective tool for measuring sleep quality over the past month, was not well suited to measuring the effectiveness of insomnia treatment. In future studies, we should consider measuring insomnia severity using the insomnia severity index. Additionally, a single‐item sleep measurement for flexibility and class repetition may also be an option. However, although this study was not focused on the mechanism underlying rTMS treatment, it is an important issue that requires further research. Our assessment of the riskiness of drug use in this study was inadequate, and prospective written documentation of potential side effects, Specifically, choose zolpidem combined with clonazepam increases the risk of epilepsy and falls in elderly patients and requires special attention, should be undertaken in future trials.

## AUTHOR CONTRIBUTIONS

HZ and CH conceived and designed the study. HY, QZ, JY, and QL performed the study and collected materials. HZ and HQ analyzed the results. HZ wrote the manuscript. HY and CH helped coordinate the study and reviewed the manuscript. All authors contributed to the article and approved the submitted version.

## FUNDING INFORMATION

This work was supported by the Introduced Project of the Suzhou Clinical Medical Expert Team (number SZYJTD201725) and Suzhou Science and Tehchnology Bureau (SYSD2020044). The funding agencies did not contribute to the experimental design or conclusions.

## CONFLICT OF INTEREST STATEMENT

The authors declare that the research was conducted in the absence of any commercial or financial relationships that could be construed as a potential conflict of interest.

## Data Availability

Data are available upon reasonable request. Some or all data generated or used during the study are available from the corresponding author by request (Hui Zheng; zh.dmtr@gmail.com).
